# Concurrent colonic mucosa-associated lymphoid tissue lymphoma and adenoma diagnosed after a positive fecal occult blood test: a case report

**DOI:** 10.1186/s13256-016-0810-1

**Published:** 2016-01-27

**Authors:** Pei-Chiang Lin, Jinn-Shiun Chen, Po Deng, Chih-Wei Wang, Chiung-Huei Huang, Reiping Tang, Jy-Ming Chiang, Chien-Yuh Yeh, Pao-Shiu Hsieh, Wen-Sy Tsai, Sum-Fu Chiang

**Affiliations:** 1 Division of Colon and Rectal Surgery, Lin Shin Hospital, Taichung, Taiwan; 2 Division of Colon and Rectal Surgery, Chang Gung Memorial Hospital, 5, Fu-Hsing Street, Kuei-Shan Hsiang, Tao-Yuan, Linkou, Taiwan; 3 Division of Hematology-Oncology, Department of Internal Medicine, Chang Gung Memorial Hospital, Linkou, Taiwan; 4 Department of Pathology, Chang Gung Memorial Hospital, Linkou, Taiwan; 5 Division of Endocrinology and Metabolism, Department of Internal Medicine, Chang Gung Memorial Hospital, Linkou, Taiwan; 6 Chang Gung University College of Medicine, Linkou, Taiwan

**Keywords:** Colon lymphoma, Endoscopic mucosal resection, Mucosa-associated lymphoid tissue lymphoma

## Abstract

**Background:**

Colonic lymphoma is an uncommon presentation of extranodal lymphoma. Colonic mucosa-associated lymphoid tissue lymphoma is a different entity from gastric mucosa-associated lymphoid tissue lymphoma, and very rare. The presentation and management of colonic mucosa-associated lymphoid tissue are highly variable in the literature.

**Case presentation:**

We report the case of a 59-year-old Taiwanese man who underwent a colonoscopy after a positive test for fecal occult blood. His past history included hypertension and hyperthyroidism. The colonoscopy revealed an adenomatous polyp and mucosa-associated lymphoid tissue lymphoma. We successfully performed a polypectomy and endoscopic mucosal resection. The lymphoma was staged according to the Ann Arbor system modified by Musshoff as E-I. Our patient showed no lymphoma recurrence over a 3-year follow-up.

**Conclusions:**

Endoscopic mucosal resection for colonic mucosa-associated lymphoid tissue lymphoma without disseminated disease may be feasible. We successfully used colonoscopic treatment without adjuvant therapy to treat early-stage pathogen-free colonic mucosa-associated lymphoid tissue lymphoma.

## Background

The gastrointestinal tract is the most common site for extranodal lymphomas, with the colon being the least involved area [1, 2]. Colon lymphomas account for 15–20 % of gastrointestinal lymphomas [1], 1.4 % of all non-Hodgkin’s lymphomas [3], and 1 % of all colorectal malignancies [4]. Mucosa-associated lymphoid tissue (MALT) lymphoma represents the third most common non-Hodgkin’s lymphoma [5], and one of the two most common types of gastrointestinal non-Hodgkin’s lymphoma [2]. Unlike gastric MALT lymphoma, colonic MALT lymphoma is very rare. The presentation of colonic MALT lymphoma is highly variable, and its management is not clear [6–11]. We treated a male patient with asymptomatic colonic MALT lymphoma, which was successfully resected by colonoscopy.

## Case presentation

Our patient was a 59-year-old Taiwanese, well-nourished man with a history of hypertension for 4 years and hyperthyroidism for one more year that were well controlled. He was a heavy smoker (1–2 packs per day) for 30 years, and had only quit for a few months. He had received a lumbar laminectomy to treat spinal stenosis 6 years previously. He had no relevant family history. He had developed thyrotoxic exophthalmos 2–3 months prior to presentation and received steroid pulse therapy for compressive optic neuropathy. The symptoms were relieved for 2–3 months after the steroid therapy.

**Fig. 1:**
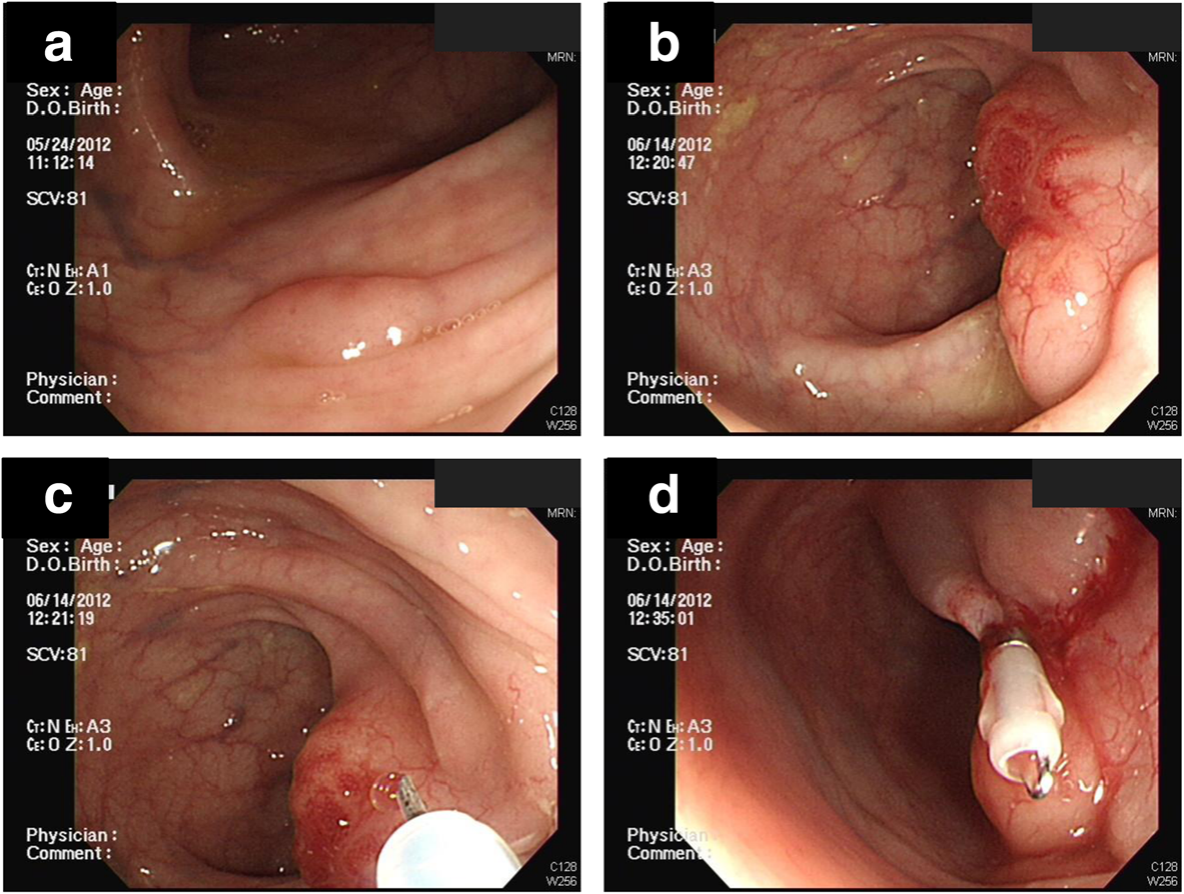
Complete colonoscopy revealed a flat polyp located 35 cm from the anal verge (**a**). Pathology identified it as an adenomatous polyp. Another 2.0 cm polypoid polyp was found 25 cm from the anal verge (**b**). It was polypoid with a wide base, slightly irregular border, and an irregular vascular pattern with mild inflammatory changes. We removed the second lesion (shown in b) by endoscopic mucosal resection (**c**), followed by the application of one hemoclip for wound closure (**d**)

He visited our clinic after a positive fecal occult blood test (132 ng/mL) but reported no change to his bowel habits. A physical examination revealed bilateral eyes proptosis and mild obesity. Complete colonoscopy revealed one flat polyp, 0.3 cm in size, located 35 cm from his anal verge, and another polypoid polyp with a wide base, 2.0 cm in size with a slightly irregular border, located 25 cm from his anal verge. The smaller polyp was removed by polypectomy. We biopsied the larger polyp because of its wide base. A pathological examination of the specimen revealed it was a hyperplastic polyp. We did not perform an endoscopic ultrasound because we intended to directly remove the polyp. The polyp was then removed by endoscopic mucosal resection followed by clipping (Fig. 1). There were no unexpected events after the procedure.

**Fig. 2:**
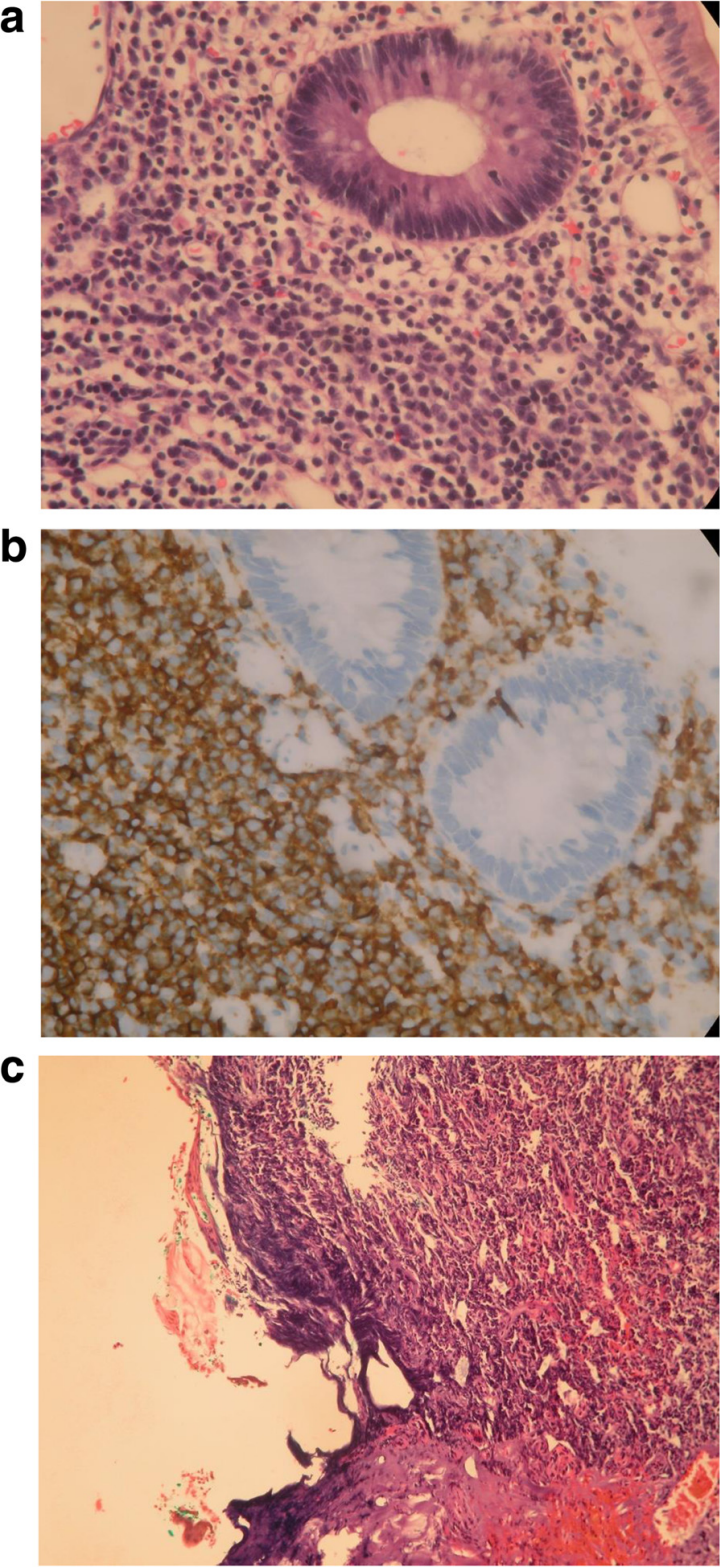
Pathology of the larger polypoid polyp revealed polypoid colonic mucosa with atypical lymphoid cells infiltrating the lamina propria (**a**). An immunohistochemical study found that the specimen was positive for CD20, CD5, and Bcl-2, and negative for CD10 and cyclin D1, which supported the diagnosis of extranodal marginal zone lymphoma of mucosa-associated lymphoid tissue lymphoma type (**b**). The resected specimen is positive for lymphoma cells, which are present at the cauterized margin (left side of the figure; hematoxylin and eosin stain, 100×) (**c**)

A pathological examination of the removed polyps identified the smaller polyp as adenomatous and the larger one as a MALT lymphoma, with a polypoid colonic mucosa and atypical lymphoid cells infiltrating the lamina propria (Fig. 2a). An immunohistochemical study demonstrated that the larger polyp was positive for CD20, CD5, and Bcl-2, and negative for CD10 and cyclin D1 (Fig. 2b). These results supported our diagnosis of extranodal marginal zone lymphoma of MALT type. However, when reviewed by a pathologist, the margins of the endoscopic mucosal resection specimen were found to be positive (cauterized margin with lymphoma cells; Fig. 2c), although it was free on gross examination.

Our patient was transferred to the hematologist’s clinic, and further studies revealed no disease dissemination. A physical examination revealed no palpable lymph nodes, no petechiae, and no hepatosplenomegaly. A computed tomography scan revealed no metastatic tumors, nor enlarged lymph nodes. A bone marrow biopsy of his right iliac bone revealed small aggregates of small lymphoid cells but an immunohistochemical study did not suggest any MALT lymphoma involvement. An otorhinolaryngologist found no abnormalities in his ear drums, nose, oral cavity, nasopharynx, or vocal cord. The tumor stage was E-I according to the Ann Arbor staging system modified by Musshoff [12].

Our patient had regular follow-up appointments with the hematologist and proctologist without any signs of lymphoma recurrence for 3 years. Two years after his initial presentation, a colonoscopy revealed a transverse colon adenoma and a rectal hyperplastic polyp. The previous site of the MALT lymphoma was free of tumor, and no other evidence of MALT lymphomas was found. A polypectomy was performed with no unexpected events.

According to his history and throughout his clinical course, our patient had no symptoms or signs of a peptic ulcer. We did not perform a panendoscopy nor test for *Helicobacter pylori*. There were no other symptoms or signs compatible with autoimmune diseases or viral infection. We did not test for Epstein-Barr virus (EBV). During the last 2 years, he has had no epigastralgia, no acid reflux, and no tarry stool.

## Discussion

MALT-type lymphoma was first defined by Isaacson and Wright in 1983 [13]. Colon lymphoma is a rare example of extranodal lymphoma. Colonic MALT lymphoma is much rarer than gastric MALT lymphoma, which is related to *H. pylori* infection [1, 2, 9]. MALT development is thought to be related to chronic antigen stimulation [7]. It comprises morphologically heterogeneous small B-cells, and typically infiltrates the epithelium [14]. Colon MALT lymphoma might be a more “benign” disease given that most cases reported in the literature were stage E-I and E-II diseases without dissemination [6–11].

The most well-known gastrointestinal lymphoma is gastric MALT lymphoma. Gastric MALT lymphoma has a strong predisposing factor, *H. pylori* infection. The prevalence of *H. pylori* infection is as high as 80 % in patients with gastric MALT lymphoma [15, 16]. However, not all patients are responsive to *H. pylori* eradication [17, 18]. Furthermore, the recurrence of gastric MALT lymphoma is accompanied by *H. pylori* infection in only 0–57 % of patients [17, 19]. Asano *et al*. reviewed *H. pylori*-negative gastric MALT lymphomas and suggested that possible mechanisms include antibiotic eradication for non-*H. pylori* bacteria and the immunomodulatory effect of clarithromycin [20].

Other associated diseases, such as EBV infection and autoimmune diseases, have also been reported in the literature [21]. Kaneko *et al*. reported the resemblance between tonsil MALT lymphoma and EBV-related tonsillar hyperplasia [21]. These two diseases can only be differentiated by immunohistochemical studies. MALT lymphomas of sites other than the stomach have also been reported. They include lung [22], tonsil [21], and ocular adnexa [23]. Most of them these lymphomas had a benign or pre-malignant nature with an excellent prognosis [22, 23].

Unlike gastric MALT lymphoma, colon MALT lymphoma does not have any obvious predisposing factors. Although chronic antigen stimulation is a hypothetical etiology [7], it was not present in most reported colon MALT lymphomas. Several cases with MALT lymphoma received and responded to *H. pylori* eradication [7, 10]. Our patient had a history of hyperthyroidism and exophthalmos, but associations between other diseases and colon MALT lymphoma had not been reported in the literature until 2–3 years ago. Jain *et al*. reported a MALT lymphoma of the cecum presenting as an acute bowel obstruction; however, no *H. pylori* nor other etiologies could be identified [24]. Terada reported the case of an 18-year-old man with a 2-year history of ulcerative colitis who developed MALT lymphoma [25]. His diagnosis was made by colonoscopic biopsy, and he only received treatment for his ulcerative colitis after that [25].

The presentation and management of colonic MALT lymphoma are still highly variable. The symptoms include a positive test for fecal occult blood, constipation, abdominal pain, gastro-intestinal bleeding, and tenesmus, or it may be asymptomatic [6–11]. Management includes local resection, colectomy, *H. pylori* eradication, chemotherapy, and radiotherapy [6–8, 10, 11]. To the best of our knowledge, our patient’s case is the first report of successful management by colonoscopy for MALT lymphoma. No antibiotic eradication was applied. Our patient has one of the longest follow-up times without recurrence (3 years) reported in the literature [6]. However, the rarity of colon MALT lymphomas means that more cases are required for comparison.

## Conclusion

Although more and more MALT lymphomas have been reported, colon involvement is still relatively rare. We report the case of a patient with a colonic MALT lymphoma with previous hyperthyroidism. Our patient was asymptomatic and underwent endoscopic mucosal resection without antibiotic eradication for a stage E-I tumor. He attended regular follow-up appointments without lymphoma recurrence for 3 years. A “wait and see” policy for resectable stage E-I colonic MALT lymphoma without pathogens seems to be a reasonable course of action. However, more studies about standard management for the disease are needed.

## Consent

Written informed consent was obtained from the patient for publication of this case report and any accompanying images. A copy of the written consent is available for review by the Editor-in-Chief of this journal.
